# Brazilian headache registry: methods and preliminary data of the pilot study

**DOI:** 10.1055/s-0043-1771175

**Published:** 2023-08-21

**Authors:** Vanise Grassi, Mauro Eduardo Jurno, Alan Christmann Fröhlich, Carlos Roberto de Mello Rieder, Elder Machado Sarmento, Júlia Kássia Pereira, Leonardo Lima Silva, Liselotte Menke Barea, Luiz Ernesto Besen Poli, Luiz Paulo Queiroz, Marcelo Cedrinho Ciciarelli, Mario Fernando Prieto Peres, Pedro Augusto Sampaio Rocha Filho, Rebeca Veras de Andrade Vieira, Renata Gomes Londero, Fernando Kowacs

**Affiliations:** 1 Hospital Moinhos de Vento, Porto Alegre RS, Brazil.; 2 Fundação Universidade Federal de Ciências da Saúde de Porto Alegre, Porto Alegre RS, Brazil.; 3 Hospital São Lucas da PUCRS, Porto Alegre RS, Brazil.; 4 Fundação José Bonifácio Lafayette de Andrada, Barbacena MG, Brazil.; 5 Fundação Hospitalar do Estado de Minas Gerais, Barbacena MG, Brazil.; 6 Serviço de Neurologia e Neurocirurgia de Passo Fundo, Passo Fundo RS, Brazil.; 7 Universidade Federal Fluminense, Rio de Janeiro RJ, Brazil.; 8 Universidade Federal de Santa Catarina, Florianópolis SC, Brazil.; 9 Faculdade de Medicina, Centro Universitário Barão de Mauá, Ribeirão Preto SP, Brazil.; 10 Hospital Israelita Albert Einstein, São Paulo SP, Brazil.; 11 Universidade Federal de Pernambuco, Recife PE, Brazil.; 12 Universidade de Pernambuco, Hospital Universitário Oswaldo Cruz, Recife PE, Brazil.; 13 Universidade do Vale do Rio dos Sinos, São Leopoldo RS, Brazil.; 14 Hospital de Clínicas de Porto Alegre, Porto Alegre RS, Brazil.

**Keywords:** Headache, Migraine Disorders, Routinely Collected Health Data, Epidemiology, Cefaleia, Transtornos de Enxaqueca, Dados de Saúde Coletados Rotineiramente, Epidemiologia

## Abstract

**Background** Evaluation and treatment of primary and secondary headaches is a global public health challenge. Recognizing the epidemiological impact of headaches, a group of researchers linked to the Brazilian Headache Society proposed the Brazilian Headache Registry and drew up its initial protocol.

**Objective** Here we describe the methods and preliminary data obtained from the pilot study.

**Methods** This was a multicenter longitudinal observational study conducted between September 2020 and August 2021. Prospective data were collected in three specialist centers for headache care in states in southern and southeastern Brazil. Patients aged 18 years or older who sought care for headache in tertiary centers and who agreed to participate in the study, were considered eligible.

**Results** Sixty-six patients were included in the pilot study: 43 (65%) from Rio Grande do Sul state and 23 (35%) from Minas Gerais state. Overall, 90% were female, and the subjects' mean age was 38.2 ± 11.2 years. Primary headaches accounted for 85.3% of the diagnoses made. Among secondary headaches, medication overuse headache was the most frequent type (7.1%).

**Conclusions** The pilot study showed the feasibility of the research protocol developed for tertiary centers. The Brazilian Headache Registry will form a source of longitudinal data with the aim of contributing to better characterization of the various phenotypes of patients with primary and secondary headaches, and to detailing the use of health resources and identifying predictors of better clinical outcomes.

## Resumo

**Antecedentes** A avaliação e o tratamento das cefaleias primárias e secundárias são um desafio global de saúde pública. Reconhecendo o impacto epidemiológico das cefaleias, um grupo de pesquisadores vinculados à Sociedade Brasileira de Cefaleia propôs a criação de um Registro Brasileiro de Cefaleia e elaborou seu protocolo inicial.

**Objetivo** Nesta publicação descrevemos os métodos e dados preliminares obtidos a partir do estudo piloto.

**Métodos** Trata-se de um estudo prospectivo observacional longitudinal multicêntrico, realizado entre setembro de 2020 e agosto de 2021. Foram coletados dados em três centros especializados no atendimento de cefaleia, em estados da região sul e sudeste do Brasil. Pacientes com idade igual ou superior a 18 anos que procuraram os centros terciários por queixa de cefaleia e concordaram em participar do estudo foram considerados elegíveis.

**Resultados** Sessenta e seis pacientes foram incluídos no estudo piloto, 43 (65%) do Rio Grande do Sul e 23 (35%) de Minas Gerais. Da amostra total, 90% eram do sexo feminino e a idade média dos sujeitos foi de 38,2 ± 11,2 anos. As cefaleias primárias representaram 85,3% dos diagnósticos realizados. Entre as cefaleias secundárias, a cefaleia por uso excessivo de medicamentos foi a mais frequente (7,1%).

**Conclusões** O estudo piloto evidenciou a viabilidade do protocolo de pesquisa desenvolvido para centros terciários. O Registro Brasileiro de Cefaleia constituirá uma fonte de dados longitudinais com o objetivo de contribuir para melhor caracterização dos diversos fenótipos de pacientes com cefaleias primárias e secundárias, detalhar o uso de recursos de saúde e identificar preditores de melhores desfechos clínicos.

## INTRODUCTION

In Brazil, it has been estimated that seven out of every ten people suffer from some type of headache. 1 Evaluation and treatment of primary and secondary headaches often involve non-evidence-based approaches, which is a persistent public health problem. 2 3 This scenario is not exclusive to Brazil. The Global Burden of Disease has estimated the impacts of more than 300 pathological conditions and injuries. 4 Functional disability, the most important parameter for estimating the impact of primary headaches, is evaluated in this study through the number of years lived with disability. 5 This measure highlighted the burden of primary headaches, especially migraine, which is always ranked among the top ten causes of disability worldwide. 4 5

Recognizing the epidemiological impact of headaches and the importance of qualifying the care for patients affected by them in our country, a group of researchers linked to the Brazilian Headache Society developed a proposal for the Brazilian Headache Registry (Registro Brasileiro de Cefaleia – REBRACEF). Clinical registries have gained importance as strategies to improve healthcare services. They allow evaluation of factors that influence prognosis and quality of life in relation to a given condition, providing measurements of the quality of care and possible disparities between regions or subpopulations. They also enable evaluation of the effectiveness and safety of the treatments implemented, through providing data on clinical results, experience and patient satisfaction, thus contributing to assessments of the healthcare system and current public policies. 6

REBRACEF was designed as a prospective multicenter clinical register of patients diagnosed with primary and secondary headaches who were seen at specialist tertiary centers for headache treatment. Among the specific objectives of this study, we sought to define the sociodemographic profile of patients attended due to complaint of headache at specialist centers; investigate the presentation characteristics and clinical evolution of primary and secondary headaches; investigate the personal, functional and economic impact of the different headache types; evaluate the effectiveness of the multiple treatments indicated, while monitoring its safety, direct and indirect costs, and risks and benefits in a real-life context; identify the factors that influence the prognosis and quality of life of these patients, including clinical and psychiatric comorbidities.

Here we describe the development of the protocol and its application in the pilot study.

## METHODS

The Brazilian Headache Society appointed a committee with the purpose of creating the registry protocol. The committee selected a group of experts, from five different states in Brazil, chosen for their expertise in the headache field and curriculum. One of the experts (MFPP) was also a representative of the patient advocates association (ABRACES – Associação Brasileira de Cefaleia em Salvas e Enxaqueca). The group met periodically to discuss the study methods.

This was a multicenter prospective longitudinal and observational study, conducted between September 2020 and August 2021, as a pilot for a register that was designed to have national coverage. Due to the COVID-19 pandemic, the recruitment did not start at the same time in the three centers. Two patients were included per week, per site, avoiding to disturb the routine of the tertiary centers.

Prospective data were collected in the form of questionnaires filled out by the participants and medical staff and in the form of headache diaries. One exception to this was the inclusion of retrospective data relating to neuroimaging tests that had been performed previously.

The participants were recruited from three specialist centers for headache care in states in the southern and southeastern regions of Brazil: two centers located in Rio Grande do Sul state and one in Minas Gerais state. Patients assisted through the Brazilian National Health System or through the supplementary healthcare or private system were included. All patients aged 18 years or older who sought care for headache in tertiary centers and who agreed to participate in the study were considered eligible. The exclusion criterion was the presence of cognitive limitations regarding the understanding of the informed consent statement and/or filling out the structured questionnaire. The study protocol was approved by the research ethics committees of the coordinating and participating centers.

An initial interview was conducted after obtaining the patient's informed consent, either immediately after a consultation at a specialist outpatient clinic or through an online interview that was scheduled according to the patient preference. The initial questionnaire contained 138 questions divided into modules: *Identification data* , with 10 questions; *Sociodemographic data* , with 8 questions; *Anthropometric data* , with 2 questions; *Habits of life* , with 7 questions; *Headache,* with 36 questions; *Impact of headache,* with 13 questions; *Quality of life* , with 8 questions; *Episodic syndromes* , with 6 questions; *Comorbidities* , with 35 questions; and *Women* , with 13 questions. This last category was only applied to female patients. Standardized evaluation instruments were used in the *Impact* , *Quality of life* and *Comorbidities* modules, but the other questions were elaborated by the researchers.

The ictal impact was assessed through the Migraine Disability Assessment Scale (MIDAS) because, although this scale is more sensitive for disability screening among migraine patients, it also allows evaluation of the impact of other types of headache. 7 8 It consists of five questions about the number of days lost in the previous three months, in whole or in part, in relation to three domains: work/school, household chores and non-work-related events (family, social and leisure). The scores range from zero to 276 and are classified as grade I (scores from 0 to 5, representing absent or minimal disability), grade II (6 to 10, mild disability), grade III (11 to 20, moderate disability) and grade IV (greater than or equal to 21, severe disability). The other questions relating to the impact of headache assessed the need for and frequency of seeking care through emergency services, the need for and number of hospital overnight stays and the indications for and number of neuroimaging tests performed.

The standardized questionnaires for evaluating psychiatric comorbidities that were used were the Patient Health Questionnaire-9 (PHQ-9) and Generalized Anxiety Disorder-7 (GAD-7). The PHQ-9 is a self-administered questionnaire for screening for symptoms of depression presented in the previous two weeks, 9 which has been validated for migraine patients. 10 Each response, referring to a specific symptom, is scored from 0 (not at all) to 3 (nearly every day). The total score can thus range from 0 to 27, such that the severity of depression is graded as follows: minimal depression (scores of 0 to 4), mild depression (5 to 9), moderate depression (10 to 14), moderately severe depression (15 to 19) and severe depression (20 to 27). The impact of depressive symptoms is evaluated in a final question, in which performing daily activities is graded as no difficulty, some difficulty, very difficult and extremely difficult.

Anxiety symptoms over the previous two weeks were assessed using GAD-7, which is a self-administered scale that has also been validated for migraine patients. 11 Each of the seven responses is scored from 0 (not at all) to 3 (nearly every day), such that the final score can range from 0 to 21. The severity of anxiety disorder is then graded as follows: no anxiety symptoms (scores of 0 to 4), mild anxiety (5 to 9), moderate anxiety (10 to 14) and severe anxiety (15 to 21).

Sleep disorders were assessed using the Athens Scale 12 13 and the Epworth Scale. 14 The Athens Scale is a self-applicable instrument consisting of eight items aimed at assessing sleep induction, nocturnal awakening, early awakening, total sleep duration and sleep quality. The responses are graded from 0 to 3, thus enabling estimation of the presence and severity of the insomnia symptom over the previous 30 days. Scores from 0 to 5 indicate absence of insomnia; 6 to 9, mild insomnia; 10 to 15, moderate insomnia; and 16 to 24, severe insomnia. 13 The Epworth Scale, which is used to assess daytime sleepiness, consists of eight items graded from 0 to 3 that evaluate the possibility of falling asleep in a variety of daily situations. Scores greater than or equal to ten indicate possibly pathological daytime sleepiness. 14

Quality-of-life assessment was performed through the World Health Organization Quality of Life 8 (WHOQoL-8) scale, which is a reduced version of the WHOQoL-Bref scale that has been validated for the Brazilian population. 15 16 It consists of eight items that assess overall quality of life, satisfaction with health, ability to perform daily activities, self-esteem, satisfaction with personal relationships, housing conditions, energy and financial resources. Each question is graded from 0 to 5, such that total scores can range from 0 to 32. Higher scores correspond to a perception that the quality of life is better.

In addition to data collection from the patient, an interview was conducted with the attending medical team, after they had signed an informed consent statement and had completed their outpatient care. This consisted of a questionnaire about the diagnosis, the neurological and headache examination and the conduct adopted, with a total of 23 questions.

Quarterly follow-up interviews were conducted by telephone or online interview, according to the patient preference. Standardized contacts were repeated by email or text messages, to schedule the date of the follow-up interview. Strategies of using repeated reminders and offering alternative ways to collect data (telephone interview or online) were chosen because these are more effective for increasing participants adherence in longitudinal studies. 17 In the follow-up interview, the patient's identification data were checked, the characteristics of the headache were reviewed and the quality-of-life and impact scales were filled out. In addition, questions about treatment adherence (2 questions), adverse events (3 questions) and patient satisfaction (2 questions) were applied, in a total of 37 questions. The headache diaries that formed part of the routine of each tertiary center were also admitted into the study for patient follow-up.

The data thus collected were analyzed in the SPSS statistical software, version 20.0 (International Business Machines Corporation, Brazil). Categorical variables were presented through frequencies and percentages. Continuous variables were presented as means and standard deviations, if normally distributed; or as medians and interquartile ranges, in the case of asymmetrical distribution. For comparisons between groups, Student's t test for independent samples or the Mann-Whitney test was used, according to the distribution of the variables. The significance level of 5% was adopted in the analyses.

## RESULTS

All patients invited to participate in the study agreed to answer the initial questionnaire, after informed consent. Sixty-six patients were included in the pilot study: 43 (65%) from Rio Grande do Sul state and 23 (35%) from Minas Gerais state. Fourteen patients (21%) were excluded from the final analysis due to loss of follow-up in the first interview (6 participants/9%) or second follow-up interview (8 participants/12%). The losses of follow-up in the first interview occurred mainly in the private system, due to the research staff limitations. One patient withdrew the consent for personal reasons. The losses of follow-up in the second interview were all associated with difficulties in reaching participants by telephone, text messages or email address. The criteria for being considered loss of follow-up were three unsuccessful contact attempts ( Table 1 ).

**Table 1:** Timepoint and reasons to loss of follow-up according to the care setting (total sample, N = 66)

State	Rio Grande do Sul (N = 43)		Minas Gerais (N = 23)
City	Porto Alegre (N = 33)	Passo Fundo (N = 10)	Barbacena (N = 23)
Health Insurance	Supplementary healthcare	Private clinic	Brazilian National Health System
Loss of follow-up	3/66 (4.5%)	4/66 (6.1%)	7/66 (10.6%)
Time of loss of follow-up	First interview (N = 2) Second interview (N = 1)	First interview (N = 4)	Second interview (N = 7)
Reasons to loss of follow-up	Patient-related (N = 2) Research team-related (N = 1)	Research team-related (N = 4)	Patient-related (N = 7)

The sociodemographic data are shown in Table 2 . Overall, 90% of the subjects were female and the mean age of the sample was 38.2 ± 11.2 years. Most of the patients (53.3%) consulted in specialist centers through supplementary healthcare plans, while 31.7% did so through Brazilian National Health System and 15.0% were seen in private clinics.

**Table 2:** Sociodemographic data and baseline characteristics (N = 60)

Variables		N	%
Sex	Female	54	90.0
	Male	6	10.0
Ethnicity	White	50	83.3
	Brown	9	15.0
	Black	1	1.7
Marital status	Married	32	53.3
	Single	20	33.3
	Separated/divorced	7	11.7
	Widower	1	1.7
Education	12 years or more	43	71.7
	9 to 11 years	11	18.3
	5 to 8 years	3	5.0
	1 to 4 years	3	5.0
Occupation/profession	Employee	23	38.3
	Civil servant	7	11.7
	Self-employed	7	11.7
	Housewife/househusband	7	11.7
	Student	6	10
	Business owner/employer	4	6.7
	Retired	3	5.0
	Unemployed	3	5.0
Working in shifts	No	37	61.7
	Yes	23	38.3
Monthly family income	< US$ 200	8	13.3
	US$ 220 to US$ 1100	24	40.0
	US$ 1100 to US$ 2200	13	21.7
	US$ 2200 to US$ 4400	13	21.7
	> US$ 4400	2	3.3
Family history of headaches	First-degree family member	27	45.0
	Second-degree family member	9	15.0
	No family history of headaches	19	31.7
	Unknown	5	8.3
Age (mean/SD)	38.2 ± 11.2		
PHQ-9 (mean/SD)	8.42 ± 6.15		
GAD-7 (mean/SD)	8.75 ± 5.42		
Epworth (mean/SD)	6.65 ± 4.56		
Atenas (mean/SD)	9.1 ± 5.37		

Abbreviation: SD, standard deviation.

In the twelve months prior to the first interview, 41.7% of the patients sought care in emergency services, 18.6% required hospitalization and 59.3% reported having had at least one neuroimaging test previously. Prior to the consultation at the specialist headache outpatient clinic, 88.1% of the patients had sought care from other healthcare professionals, mostly neurologists (60.4%). Half of the patients had been diagnosed with some type of clinical or psychiatric comorbidity, among which anxiety (46.4%) and depression (35.7%) were the most prevalent. Suicidal ideation in the PHQ-9 questionnaire was identified in one patient (1.6%). The research team was instructed to immediately contact the assistant medical team to provide psychiatric/psychological support to the participant.

The attending medical team questionnaire took a mean time of 5 to 10 minutes to answer. Primary headaches predominated as the etiology of headache, corresponding to 88.4% of the diagnoses made by medical teams. Among secondary headaches, medication overuse headache was the most frequent type, accounting for 7.1% of the sample ( Table 3 ). The physical examination was described as normal for most of the patients (89.1%), and cephalic segment sensitization signs were the most frequently found in the headache examination.

**Table 3:** Diagnoses of primary and secondary headaches according to the medical team (N = 60)

Diagnoses		N	Percentage (%)
Primary headaches	Migraine without aura	31	55.4
	Migraine with aura	11	19.6
	Chronic migraine	6	10.7
	Probable migraine	2	3.6
	Infrequent episodic tension-type headache	1	1.8
	Frequent episodic tension-type headache	3	5.4
	Chronic tension-type headache	2	3.6
	Primary stabbing headache	1	1.8
	New daily persistent headache	1	1.8
Secondary headaches	Medication overuse headache	4	7.1
	Cervicogenic headache	3	5.4
	Headache attributed to chronic or recurring rhinosinusitis	1	1.8
	Headache attributed to temporomandibular disorder	1	1.8
	Headache attributed to cervical myofascial pain*	1	1.8

Note: *Diagnosis made in accordance with the Appendix of the third edition of the International Classification of Headache Disorders (ICHD-3). 18

Regarding the conduct adopted by the medical teams at the first consultation, 93.0% of the patients received a prescription for acute treatment, 82.5% for prophylactic treatment, 18.2% for transition treatment and 12.5% for rescue treatment. Tricyclic antidepressants and beta-blockers were the drugs most indicated for prophylactic treatment. Gradual reduction/suspension of overuse of symptomatic drugs was indicated for 34.1% of the patients. Complementary tests (laboratory tests or neuroimaging) were recommended for only 3.3% of the patients.

In the three-month follow-up questionnaire, in a 10 to 15-minute interview, 78.8% of the patients declared that they were fully maintaining the prescribed treatment, while 9.6% were only maintaining it partially (only drugs for symptomatic or prophylactic use). The reasons for discontinuation of treatment that were most described were mild adverse events and absence of therapeutic response. The majority (82.7%) of the patients reported that they were totally or partially satisfied with the treatment. Among the dissatisfied patients, the main reason for dissatisfaction, reported by 11.5% of them, was the desire not to feel headaches anymore. The comparative analysis between the first and second interviews are shown in Table 4 .

**Table 4:** Comparative analysis between initial and quarterly interviews (N = 52)

		Initial	Quarterly	p
Headache intensity	Severe	69.2%	28.8%	<0.001*
	Moderate	28.9%	55.8%	
	Mild	1.9%	13.5%	
	Missing data	0	1.9%	
MIDAS, median (IQR)		14.0 (4-35)	10.5 (3-30)	0.502*
WHOQol-8, mean ± SD		19.58 ± 5.21	21.4 ± 5.32	0.007**
Headache frequency	Low frequency (1-7 days per month)	40.4%	61.5%	0.27***
	High frequency (8-14 days per month)	13.5%	19.2%	
	Chronic (≥ 15 days per month)	30.8%	15.4%	
	Missing data	15.3%	3.9%	
Medication overuse	Yes	59.1%	36%	0.005***
	No	40.9%	64%	

Abbreviations: IQR, interquartile range; SD, standard deviation. Note: *Wilcoxon test; **Student test; ***Mac Nemar.

## DISCUSSION

REBRACEF was developed with the aim to become a repository for clinical data of national scope, such that it will document the presentation, evolution and determinants of different types of headache in a developing country. REBRACEF is, to the best of the authors' knowledge, the first specific register of patients affected by primary and secondary headaches in South America.

The idea of collecting data from headache patients in an organized multicenter registry is not new 19 20 21 22 23 24 and there are many examples of analyzes that emerged from clinical registries. 25 26 27 28 The Brazilian protocol shares similarities with other registries regarding sociodemographic data collected, headache features investigated, patient-reported outcomes measured and comorbidities evaluated. 19 20 21 22 23 24 To the best of the authors' knowledge, REBRACEF is the first registry to investigate suicidal ideation and to offer a support system for the patient at risk. The main operational limitation of our registry is the impossibility of using an electronic headache diary, as performed in other studies. 19 20 21 22 23 By including participants with primary and secondary headaches, REBRACEF is able to identify patients with rare conditions from different regions of the country and contribute to a better description and management of these disorders.

The development of a unified clinical registry encompassing the Brazilian territory is extremely challenging, considering the great regional disparities, especially regarding socioeconomic conditions, regional peculiarities of the language and communication strategies needed. Some adjustments were made from the pilot study, considering these disparities. The decision to maintain the initial data collection on paper forms and to record additional numbers for telephone contact was taken from the moment it was identified that almost half of the patients seen through the Brazilian National Health System did not provide an email address. The paper headache diary was also a decision taken in the direction of making the registry more inclusive.

The clinical registries must ideally include a significant number of participants for a prolonged period to achieve meaningful results, which requires a great commitment from participating centers. This includes the need for dedicated staff, software and analytical experts. In addition, registries must generally rely on a cadre of committed volunteer physician leaders who develop and periodically revise data elements and oversee registry operations. Considering the loss of follow-up in the private setting, where only one investigator was involved, it is recommended that the research team involves at least four members, including junior and senior researchers.

REBRACEF has the aim of including and monitoring a broad and representative sample of the population with primary and secondary headaches. The conclusions from this pilot study should therefore be viewed with caution, considering the small sample size and the low percentage of patients attended through the public healthcare system. This percentage may explain the demographic characteristics of the pilot study that differed from those of the general Brazilian population, such as the high level of education and family income and the low presence of non-Caucasian individuals in the study sample. The next phase of the study should include a sample more representative of the Brazilian population, considering that tertiary services in other regions of the country and/or dedicated to the care of Brazilian National Health System patients should be included.

The predominance of conditions that can be clinically diagnosed, especially migraine and its subtypes, contrasts with the high percentage of patients undergoing investigation through complementary tests, thus corroborating data from previous studies. 2 3 The fact that, in more than half of the cases, care had previously been provided by neurologists indicates that, in addition to the gap in generalist medical education, another possible barrier to care for patients with headache is the scarcity of specialists trained to provide adequate care for patients complaining of headache. 29

The clinical data for this pilot study were obtained from specialist headache centers, where patients affected by more severe pathological conditions and with higher degrees of associated disability are frequently seen. Although these characteristics do not invalidate the clinical registers from specialist centers, the data obtained cannot be generalized to other care scenarios. Since no patient with trigeminal autonomic headache was included in the pilot study, no information on the applicability of the questionnaire in this context was obtained.

In conclusion, the pilot study showed the feasibility of the protocol developed for tertiary centers, with the commitment of most patients both in filling out the forms and in the follow-up interview. The majority of the patients treated at these specialist headache centers had some form of primary headache and reported satisfaction with the results at the first three-month follow-up. Considering that these are pathological conditions that essentially involve a clinical diagnosis and do not require access to sophisticated technological resources, it can be assumed that better training for physicians and for general neurologists can assuage a large proportion of patients' demands for treatment at tertiary centers.

Future perspectives include the identification of the most relevant aspects in the care of patients with headache in specialist centers and the implementation of new phases of the study including secondary and primary care scenarios, with simplified protocols. REBRACEF will be a source of longitudinal data with the aim of contributing to better characterization of the various phenotypes of patients with primary and secondary headaches, thereby detailing the use of healthcare resources and identifying predictors for better clinical outcomes.
